# Complex network of eye movements during rapid automatized naming

**DOI:** 10.3389/fnins.2023.1024881

**Published:** 2023-03-31

**Authors:** Hongan Wang, Fulin Liu, Dongchuan Yu

**Affiliations:** ^1^Key Laboratory of Child Development and Learning Science of Ministry of Education, School of Biological Science and Medical Engineering, Southeast University, Nanjing, China; ^2^Henan Provincial Medical Key Lab of Child Developmental Behavior and Learning, The Third Affiliated Hospital of Zhengzhou University, Zhengzhou, Henan, China

**Keywords:** developmental dyslexia, rapid automatized naming, eye tracking, time series, complex network

## Abstract

**Introduction:**

Although the method of visualizing eye-tracking data as a time-series might enhance performance in the understanding of gaze behavior, it has not yet been thoroughly examined in the context of rapid automated naming (RAN).

**Methods:**

This study attempted, for the first time, to measure gaze behavior during RAN from the perspective of network-domain, which constructed a complex network [referred to as *gaze-time-series-based complex network* (GCN)] from gaze time-series. Hence, without designating regions of interest, the features of gaze behavior during RAN were extracted by computing topological parameters of GCN. A sample of 98 children (52 males, aged 11.50 ± 0.28 years) was studied. Nine topological parameters (i.e., average degree, network diameter, characteristic path length, clustering coefficient, global efficiency, assortativity coefficient, modularity, community number, and small-worldness) were computed.

**Results:**

Findings showed that GCN in each RAN task was assortative and possessed “small-world” and community architecture. Additionally, observations regarding the influence of RAN task types included that: (i) five topological parameters (i.e., average degree, clustering coefficient, assortativity coefficient, modularity, and community number) could reflect the difference between tasks N-num (i.e., naming of numbers) and N-cha (i.e., naming of Chinese characters); (ii) there was only one topological parameter (i.e., network diameter) which could reflect the difference between tasks N-obj (i.e., naming of objects) and N-col (i.e., naming of colors); and (iii) when compared to GCN in alphanumeric RAN, GCN in non-alphanumeric RAN may have higher average degree, global efficiency, and small-worldness, but lower network diameter, characteristic path length, clustering coefficient, and modularity. Findings also illustrated that most of these topological parameters were largely independent of traditional eye-movement metrics.

**Discussion:**

This article revealed the architecture and topological parameters of GCN as well as the influence of task types on them, and thus brought some new insights into the understanding of RAN from the perspective of complex network.

## 1. Introduction

Rapid automatized naming (RAN) tasks (Bowers and Wolf, 1993; Kail et al., 1999; Wiig et al., 2000; Stainthorp et al., 2010; American Psychiatric Association, 2013; Decker et al., 2013; Georgiou et al., 2013; Powell et al., 2014; Hjetland et al., 2017; Akhand et al., 2019; Ullman et al., 2020) have been developed to assess the ability to name a serially presented list of numbers, letters, words, colors, or objects as rapidly as possible. RAN abilities, in combination with other cognitive skills (e.g., phonological awareness, short-term memory, letter knowledge, and vocabulary), have been extensively interpreted, and they characterized the features of both reading-related behavior and developmental dyslexia (Goswami, 2015; Åvall et al., 2019; Georgiou and Parrilla, 2020; Mcweeny et al., 2021). The eye-tracking technique (Armstrong and Olatunji, 2012; Lai et al., 2013; Chita-Tegmark, 2015; Frazier et al., 2017) would be a promising tool to capture the visual cognitive features of the gaze behavior during RAN because it is simple to use, objective, and suitable for usage with all ages from infancy to adulthood. In particular, it can be utilized to monitor the focus locations sequentially and document the critical ocular activities during RAN. Even though several studies (Jones et al., 2008, 2016; Kuperman and Van Dyke, 2011; Pan et al., 2013; Hogan-Brown et al., 2014; Gordon and Hoedemaker, 2016; Kuperman et al., 2016; Silva et al., 2016; Nayar et al., 2018, 2021; Akhand et al., 2019; Araújo et al., 2021; Wang et al., 2022) have explored the characteristics of gaze behavior during RAN, only a few of them have ever examined whether and how gender, age, and task type alter these characteristics simultaneously. Additionally, researchers have not fully explored gaze behavior during a Chinese adaptation of RAN in order to broaden the application of RAN to developmental dyslexia in Chinese.

Regarding the types of eye-movement metrics (Armstrong and Olatunji, 2012; Lai et al., 2013; Chita-Tegmark, 2015; Frazier et al., 2017), there are three widely used categories, namely, fixation-related metrics, saccadic-related metrics, and scan-path (or fixation sequence)-related metrics. Fixation-related metrics are usually associated with the eye pauses within regions of interest (ROIs), where cognitive processing is believed to occur; saccadic-related metrics are associated with rapid movements that occur between fixation events; and scan-path-related metrics are often employed to examine sequencing of cognitive allocation across a stimulus. It should be noted that these traditional eye-movement metrics typically rely on the definition (or prior knowledge) of ROIs and require setting the spatial range of these ROIs manually before statistical analysis.

Recent studies (Constantino et al., 2017; Del Bianco et al., 2021; Hedger and Chakrabarti, 2021; Nayar et al., 2022) proposed the idea of treating the eye-tracking data as a time series, allowing us to track the moment-by-moment changes in gaze behavior during cognitive tasks. Although this idea has been hailed as a significant advancement in eye-tracking analysis, it has not yet been thoroughly examined in the context of RAN (Wang et al., 2022). Additionally, the performance would be further enhanced if a more potent time series analysis technique can be adopted from the perspective of non-linear dynamics. For instance, Wang et al. (2022) showed an entropy-based method to measure gaze time series during RAN, which was more sensitive to reflect small perturbations of eye movements than traditional eye-movement metrics (e.g., total time of naming).

On the contrary, the complex network analysis method (Butts, 2009; Wu et al., 2018; Zhou et al., 2018) has been well-documented and has shown a powerful idea: first, construct a complex network in different contexts by using the proper definition of nodes and edges; then, calculate topological properties of the complex network from the viewpoint of graph theories; and finally, reveal the between-group difference of topological properties or even identify diagnostic features (biomarkers). As a promising research direction of complex network analysis, some scientists have insightfully explored the feasibility of the method of converting time series into a complex network (Zhang and Small, 2006; Lacasa et al., 2012). As an illustration, a visibility graph algorithm (Lacasa et al., 2012) has been proposed to map one-dimensional time series into a complex network. Its effectiveness and potential utility have been confirmed due to the fact (Lacasa et al., 2012) that the structure of time series can be conserved in the network topology: periodic time series convert into regular networks, random time series convert into random networks, and fractal time series convert into scale-free networks. Consequently, as a main motivation, this study sought to examine (i) whether the gaze time series during RAN could be converted into a complex network by using the proper definition of nodes and edges and (ii) whether and how the network's properties could capture the fundamental characteristics of the gaze behavior during RAN.

Taken together, this study attempted, for the first time, to measure the gaze behavior during RAN from the perspective of the network domain, which constructed a complex network [referred to as *gaze-time-series-based complex network* (GCN)] from gaze time series. In this way, network-domain evaluation of the gaze behavior during RAN was conducted by computing the topological properties of GCN. The strengths of the suggested technique were 2-fold. First, it conducted a network-domain evaluation of gaze behavior using two-dimensional time series (i.e., temporal-spatial information of eye gazes), which was different from the visibility graph approach (Lacasa et al., 2012) using one-dimensional time series. Second, it did not involve manually setting ROIs in contrast to typical eye-tracking analysis. To illustrate the feasibility of the suggested technique, this study recruited 98 children (52 boys, aged 11.50 ± 0.28 years) and conducted a network-domain evaluation of gaze behavior during RAN for each child by computing the structure and topological properties of GCN. It also discussed the potential ocular mechanisms that could explain the existence of the GCN's structure. Additionally, this study investigated the effects of various RAN tasks on the topological properties of GCN, as well as the association between these topological properties and traditional (typical) eye-movement metrics.

## 2. Materials and methods

The Southeast University Research Ethics Committee gave its approval to all study protocols and research techniques, ensuring that they adhered to the World Medical Association's Declaration of Helsinki regarding the use of humans in testing. All participating children's parents gave their informed consent, and each participant gave their verbal consent. After finishing the research, each child was given a toy that was appropriate for their age.

### 2.1. Study design and participants

This study was conducted in Sanmenxia, Henan Province, China, between September 2021 and March 2022. According to the districts' rankings of GDP per person in 2020, the districts of Sanmenxia were divided into three levels, namely, strong economic level (>90,000 RMB), medium economic level (70,000–90,000 RMB), and weak economic level (<70,000 RMB). In order to prevent bias in sample selection, this study randomly selected a district with a medium economic level and randomly selected an ordinary primary school locally from the district. This study focused on gaze behavior for only sixth-grade children to control the potential influence of age on RAN abilities. There were five classes in the sixth grade in this primary school. For each class, 11 boys and 11 girls were randomly recruited to participate in the experiment. In this way, 110 children were initially invited to participate in the current study.

Exclusion criteria were as follows: (a) children with abnormal hearing functioning (i.e., hearing threshold levels bigger than 25 dB HL) and vision functioning (i.e., naked or corrected monocular visual acuities below than 1.0); (b) children with significant sensory or motor impairment; (c) children with a history of previous neurological or psychiatric disorders; (d) children with IQ score lower than 85 or higher than 115 [normal IQ score ranges from 85 to 115; since IQ score is correlated with RAN abilities (Hogan-Brown et al., 2014; Wolff, 2014; Nayar et al., 2018, 2021), too low or high IQ score might lead to biased evaluation for the normative data of typically developing children; (e) children who had repeated a grade; (f) bilingual children; and (g) children who cannot complete the tasks or whose experimental data are incomplete. Based on inclusion and exclusion criteria, a total of 98 children (52 boys, aged 11.50 ± 0.28 years) were invited to participate in the current experiments in the end.

### 2.2. RAN procedures

**Figure 1:**
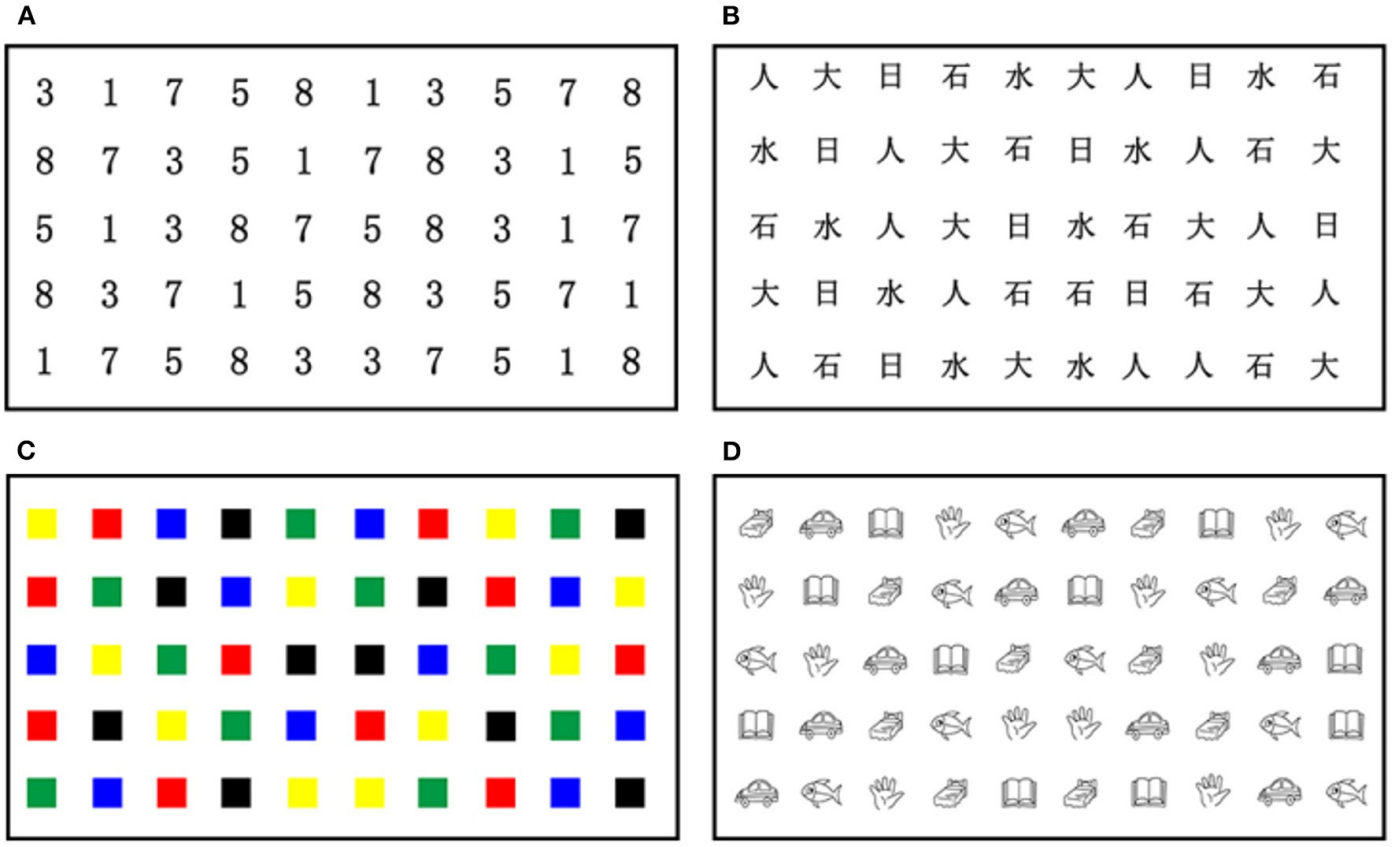
The Chinese RAN paradigm presented a 5 × 10 matrix of stimuli (e.g., numbers, Chinese characters, colors, or objects) in different tasks: **(A)** Task N-num (i.e., naming of numbers); **(B)** Task N-cha (i.e., naming of Chinese characters); **(C)** Task N-col (i.e., naming of colors); and **(D)** Task N-obj (i.e., naming of objects). RAN, rapid automatized naming.

To extend the application of RAN to developmental dyslexia in Chinese, this study employed a Chinese adaptation of RAN (C-RAN; refer to Figure 1) that substituted highly frequently used Chinese characters for English letters. As shown in Figure 1, the C-RAN paradigm in this study consisted of four tasks, namely, Task N-num (i.e., naming of numbers), Task N-cha (i.e., naming of Chinese characters), Task N-col (i.e., naming of colors), and Task N-obj (i.e., naming of objects). It should be remarked that tasks N-obj and N-col belong to non-alphanumeric RAN tasks, while tasks N-num and N-cha belong to alphanumeric RAN tasks. For each task, a 5 × 10 matrix of objects was presented, in which each matrix used five repetitions of each of the 10 different objects with the order pseudo-randomized.

Participants were tested in a quiet room. Each participant was situated between 60 and 90 cm away from the 21.5 in. TFT LCD monitor (with 1,920 × 1,080 resolution) displaying the stimuli of each RAN task shown in Figure 1. Following a standard 9-point calibration procedure, all participants were instructed to name the stimuli (numbers, Chinese characters, colors, or objects) of each RAN task as accurately and rapidly as possible from left to right in each row, and from top to bottom for all rows. To ensure comprehension of task instructions, all participants required practice by naming a 2 × 5 matrix of objects before the formal test of each RAN task. Eye movements during naming were recorded using a Tobii 4C eye tracker (90 Hz; Tobii Technology AB, Danderyd, Sweden). In order to avoid the reliability of data collection, all participants were asked not to shake their bodies (especially their heads) visibly. Participants were re-calibrated following any large movements.

To ensure the consistency and fidelity of the administration of evaluation tools, a senior expert with a professional experience of more than 8 years carried out the measures for all participating children. The senior expert had training in the administration of all tools used in this study.

### 2.3. Traditional eye-movement metrics

This study defined a “*fixation* point” as a point at which a gaze was held for at least 100 ms and gaze shifts were within a 40-pixel grid. A *saccade* can be observed if rapid movements occur between two fixations; the *saccade amplitude* can be calculated by the distance between two fixations; and *regressions* typically occur when there are backward eye movements toward previously visited items. Based on the definitions above, six traditional (typical) eye-movement metrics (i.e., average fixation duration, fixation counts, average saccade amplitude, saccade counts, regression counts, and a total time of naming) were computed as follows (Armstrong and Olatunji, 2012; Lai et al., 2013; Chita-Tegmark, 2015; Frazier et al., 2017) to evaluate gaze behavior during RAN tasks. (1) The total number of fixations can be calculated as *fixation counts*; (2) The total number of saccades can be calculated as *saccade counts*; (3) The total number of regressions can be calculated as *regression counts*; (4) *Average saccade amplitude* can be calculated by averaging the amplitude of each saccade; (5) *Average fixation duration* can be calculated by averaging duration time of each fixation; (6) The total time of completing a RAN task can be calculated as the *total time of naming*. According to earlier research (Wiig et al., 2000), the mean percentage of RAN accuracy remained constant and did not significantly change with age for typically developing (TD) individuals (aged 6–21 years) in the United States. Hence, this study focused on the total time of naming (used to measure reading fluency and speed) only and did not record the accuracy of naming during RAN (even though this parameter may be useful for the diagnosis of dyslexia).

### 2.4. Topological properties of complex network

Topological properties of the complex network can be calculated from the viewpoint of graph theories. Numerous topological properties have been proposed and can be applied in different contexts (Butts, 2009; Muldoon et al., 2016; Bassett and Bullmore, 2017; Wu et al., 2018; Zhou et al., 2018). In this study, nine topological properties were computed as follows.

(1) Average Degree can be calculated as follows:

(1) $$\begin{array}{l} MeanDegree=m/n \end{array}$$

where *m* is the total number of edges, and *n* is the total number of nodes.

(2) Network Diameter can be calculated as follows:

(2) $$\begin{array}{l} Diameter=max_{i,j}\left(d_{ij}\right) \end{array}$$

where *d*_*ij*_ is the distance between nodes *i* and *j*.

(3) Characteristic Path Length can be calculated as follows:

(3) $$\begin{array}{l} L=\frac{1}{n\left(n-1\right)}\sum_{i\neq j}d_{ij} \end{array}$$

where *n* is the total number of nodes, and *d*_*ij*_ is the distance between nodes *i* and *j*.

(4) Clustering Coefficient can be calculated as follows:

(4) $$\begin{array}{l} C=\left(\frac{1}{m}\right)\sum_{i}2E_{i}/\left[k_{i}\left(k_{i}-1\right)\right] \end{array}$$

where *m* is the total number of edges, *k*_*i*_ is the degree of the *i*-th node, and *E*_*i*_ is the number of triangles attached to the *i*-th node.

(5) Global Efficiency can be calculated as follows:

(5) $$\begin{array}{l} GE=\frac{1}{n\left(n-1\right)}\underset{i\neq j}{\sum }\left(\frac{1}{d_{ij}}\right) \end{array}$$

where *n* is the total number of nodes, and *d*_*ij*_ is the distance between nodes *i* and *j*.

(6) Assortativity Coefficient can be calculated as follows:

(6) $$AC=\frac{\sum_{i}w_{i}k_{i}/m-{\left[\sum_{i}\left(w_{i}+k_{i})/(2m\right)\right]}^{2}}{\sum_{i}(w_{i}^{2}+k_{i}^{2})/(2m)-{\left[\sum_{i}\left(w_{i}+k_{i})/(2m\right)\right]}^{2}}$$

where *m* is the total number of edges, and *w*_*i*_ and *k*_*i*_ are the degrees of the nodes at the ends of the *i*-th edge, with *i* =1, 2, …, *m*.

It has been shown (Newman, 2002, 2003) that the assortativity coefficient *AC* is the Pearson correlation coefficient for the degrees of neighboring nodes, which is supposed to have bounds *AC* ϵ[−1, 1]. In particular, a network is assortative when *AC* > 0 and disassortative when *AC* < 0 (Newman, 2002, 2003).

(7) Modularity can be calculated as follows:

(7) $$Q=(\frac{1}{2m}){\sum^{\text{​}}}_{i,j}[Aij-k_{i}k_{j}/(2m)]\delta (c_{i},c_{j})$$

where *m* is the total number of edges; *A*_*ij*_ is the adjacency matrix; *k*_*i*_ and *k*_*j*_ are the degrees of nodes *i* and *j*, respectively; *c*_*i*_ and *c*_*j*_ are the communities that nodes *i* and *j* belong to, respectively; and δ is a simple delta function that takes 1 when *c*_*i*_ equals *c*_*j*_, 0, otherwise.

It has been shown (Newman, 2004; Newman and Girvan, 2004) that *Q* ϵ [0, 1]; and the greater the modularity *Q*, the clearer the community structure.

(8) Community Number is referred to as the total number of communities when modularity Q reaches its maximal value. That is, Community Number can be calculated as follows:

(8) $$\begin{array}{l} community\;number=\mathrm{argmax}\left(Q\right) \end{array}$$

where *Q* is defined in Equation 7.

(9) Small-Worldness (i.e., small-world property) can be calculated as follows:

(9) $$SW=1-\sqrt{(\Delta_{c}^{2}+\Delta_{L}^{2})/2}$$

with $\Delta_{C}=\frac{C_{latt}-C_{obs}}{C_{latt}-C_{rand}}$ and $\Delta_{L}=\frac{L_{obs}-C_{rand}}{L_{latt}-L_{rand}}$, where *C*_*obs*_ and *L*_*obs*_ are the clustering coefficient and characteristic path length of the observed network, respectively; *C*_*latt*_ and *L*_*latt*_ are the clustering coefficient and characteristic path length of the lattice network constructed with the same number of nodes and degree distribution as the observed network, respectively; and *C*_*rand*_ and *L*_*rand*_ are the clustering coefficient and characteristic path length of the random network constructed with the same number of nodes and degree distribution as the observed network, respectively.

It has been shown (Muldoon et al., 2016) that *SW* ϵ [0, 1], and networks with a value *SW* closer to 1 will have more small-world characteristics. Additionally, Bassett and Bullmore (2017) suggested a threshold of *SW* > 0.4 for the network to be considered a small-world network but stressed that this measure should be seen as continuous, with increasing *SW* indicating an increasingly small-worldness.

### 2.5. Complex network mapped from time series of eye gazes

**Figure 2:**
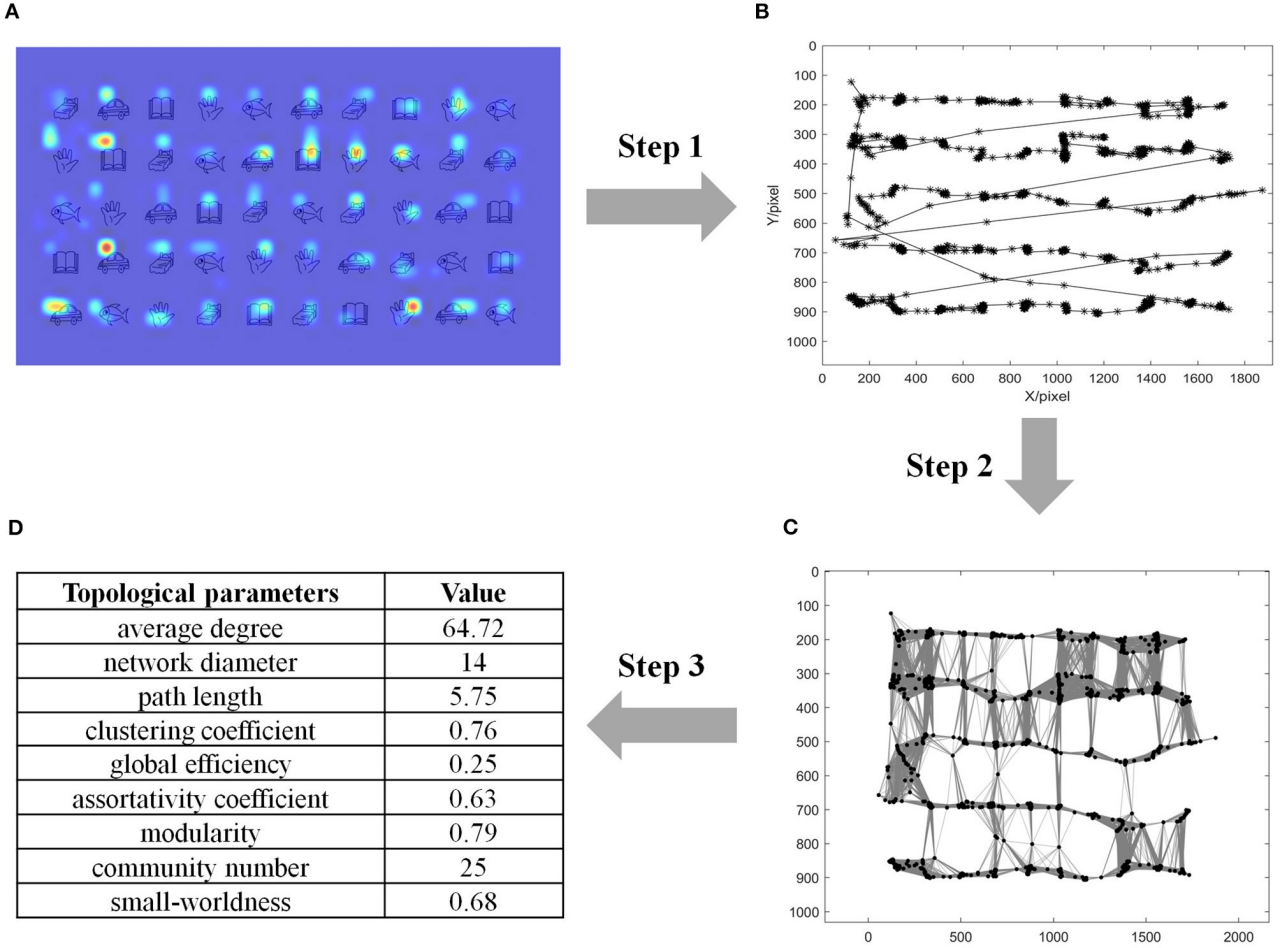
Method, involving three steps, to construct the gaze-time-series complex network (GCN) in an illustrating example of a child during a RAN task. **(A)** Heatmap of gazes in the illustrating example; **(B)** Raw spatial coordinate of eye gazes in the illustrating example, where two adjacent gazes were linked by a straight line; **(C)** The network connectivity of GCN in the illustrating example, where dots represented nodes and black lines represented connections between nodes; **(D)** Topological properties analysis and their values in the illustrating example. RAN, Rapid automatized naming.

This study sought to measure the gaze behavior during RAN from the perspective of the network domain, which constructed a complex network (referred to as GCN) from gaze time series. To better demonstrate our approach, this article used raw eye-tracking data of a child during a RAN task as an illustrating example (refer to the heatmap in Figure 2A). As depicted in Figure 2, our algorithm included the following three steps:

**Step 1 (Gaze time-series data acquisition):** Let the *m*-th gaze coordinate be represented by (*x*_*m*_, *y*_*m*_). Raw gaze coordinates were recorded at every sampling point. Figure 2B showed the trajectory of eye gazes in the illustrating example, where two adjacent gazes were linked by a straight line.

**Step 2 (Network construction):** The gaze time series was mapped into a complex network (referred to as GCN), where the *m*-th gaze was taken as the *m*-th node. The connection between nodes *w* and *m* was established according to the following rule:


(10) $$g_{mw}=\{\begin{array}{cc} 1, & d_{mw}<\theta_{mw}\\ 0, & \;otherwise \end{array}$$

where *d*_*mw*_ is the Euler's distance between nodes *w* and *m*; θ_*mw*_ is the threshold determined by the distribution of neighbors (Zhu et al., 2016) of nodes *m* and *w*. In this way, a complex network (i.e., GCN) was constructed, which was defined by the graph *G* = (*g*_*ij*_). Figure 2C visualizes the network connectivity of the GCN in the illustrated example.

**Step 3 (Topological properties analysis):** Nine parameters (i.e., average degree, network diameter, characteristic path length, clustering coefficient, global efficiency, assortativity coefficient, modularity, community number, and small-worldness) of GCN were computed for each participating child. Figure 2D shows the detailed values of these topological parameters in the illustrating example.

### 2.6. Statistical analysis

We first examined our data to determine appropriate statistical models (parametric vs. non-parametric). After confirming that our data failed to pass the normality test and variance homogeneity test, we performed a series of non-parametric Friedman rank-sum tests (Eisinga et al., 2017) for the nine topological parameters of GCN, where the effect size was measured by Kendall's *W* and was defined as small (*W* = 0.1), medium (*W* = 0.3), and large (*W* = 0.5). Additionally, for *post-hoc* multiple comparisons, we utilized the non-parametric Wilcoxon signed rank tests with the Bonferroni correction applied to *p*-values to control the false discovery rate (FDR).

To examine the independence between each of the nine topological parameters and six traditional eye-movement metrics, we carried out a series of rank-based non-parametric linear regression analyses (Hettmansperger and McKean, 2011) to obtain regression models, where each of the nine topological parameters of GCN was taken as the dependent variable; while the six traditional eye-movement metrics were taken as the independent variable in different RAN tasks. The determination coefficient *R*^2^ was used to evaluate the fitting degree of a regression model.

All statistical analysis above was conducted with R language (version 4.0.2), and the significance level α was set at 0.05. In particular, R packages “rstatix” (Kassambara, 2021) and “Rfit” (Kloke and McKean, 2012) were used.

## 3. Results

### 3.1. Topological properties changed with RAN tasks

**Figure 3:**
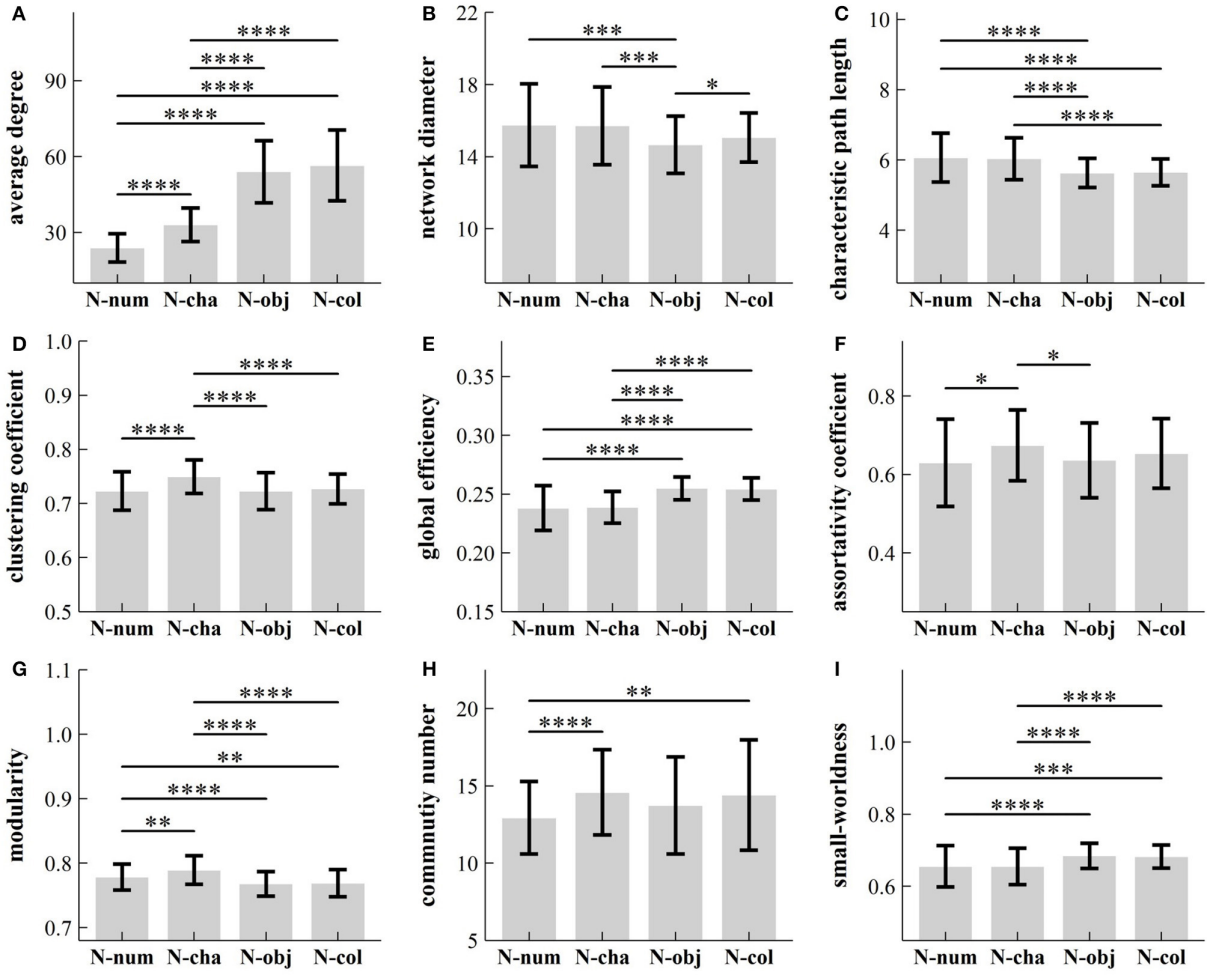
Influence of task types on different topological parameters: **(A)** average degree; **(B)** network diameter; **(C)** characteristic path length; **(D)** clustering coefficient; **(E)** global efficiency; **(F)** assortativity coefficient; **(G)** modularity; **(H)** community number; and **(I)** small-worldness. *p < 0.05; **p < 0.01; ***p < 0.001; ****p < 0.0001. RAN, rapid automatized naming; N-num, naming of numbers; N-cha, naming of Chinese characters; N-col, naming of colors; N-obj, naming of objects.

We verified that our data failed to pass both the normality test and the variance homogeneity test (*p*-values ≥ 0.05). Hence, we conducted a series of non-parametric ANOVA procedures to reveal the influence of task types on each of the nine topological properties of GCN. Table 1 summarized our results and showed that there were significant differences between four RAN tasks for the nine topological parameters [$x_{\left(3\right)}^{2}$: 11.58–265.82; *p*-values ≤ 9 × 10^−3^; Kendall's *W*: 0.04–0.904]. Additionally, we carried out the *post-hoc* test for multiple comparisons. Figure 3 summarizes our results and shows that:

(1) Average Degree: *Average degree* in task N-num was significantly lower than that in the other three RAN tasks (*p-*values < 1 × 10^−4^), while the *average degree* in task N-cha was significantly lower than that in tasks N-obj and N-col (*p-*values < 1 × 10^−4^). However, there was no significant difference between tasks N-col and N-obj in *average degree* (*Z* = 1,838, *p* = 0.23).
(2) Network Diameter: *The network diameter* in task N-num or N-cha was significantly higher than that in task N-obj (*p*-values < 1 × 10^−3^), while there was a significant difference between tasks N-obj and N-col in *network diameter* (*p* < 0.05).
(3) Characteristic Path Length: *Characteristic path length* in task N-num was significantly lower than that in tasks N-obj and N-col (*p*-values < 1 × 10^−4^), while the *characteristic path length* in task N-cha was significantly higher than that in tasks N-obj and N-col (*p*-values < 1 × 10^−4^). However, there was no significant difference between tasks N-num and N-cha (*Z* = 2,444, *p* = 1.0), as well as between tasks N-col and N-obj (*Z* = 2,186, *p* = 1.0), in *characteristic path length*.
(4) Clustering Coefficient: *The clustering coefficient* in task N-num was significantly lower than that in task N-cha (*p* < 1 × 10^−4^), while the *clustering coefficient* in task N-cha was significantly higher than that in tasks N-obj and N-col (*p*-values < 1 × 10^−4^). However, there was no significant difference between other tasks in the *clustering coefficient* (*p*-values > 0.05).
(5) Global Efficiency: *Global efficiency* in task N-num was significantly lower than that in tasks N-obj and N-col (*p*-values < 1 × 10^−4^), while *global efficiency* in task N-cha was significantly lower than that in tasks N-obj and N-col (*p*-values < 1 × 10^−4^). However, there was no significant difference between tasks N-num and N-cha (*Z* = 2,622, *p* = 1.0), as well as between tasks N-col and N-obj (*Z* = 2,653, *p* = 1.0), in *global efficiency*.
(6) Assortativity Coefficient: *The assortativity coefficient* in task N-num was significantly lower than that in task N-cha (*p* < 0.05), while the *assortativity coefficient* in task N-cha was significantly higher than that in task N-obj (*p* < 0.05). However, there was no significant difference between tasks N-col and N-obj in the *assortativity coefficient* (*p* > 0.05).
(7) Modularity: *Modularity* in task N-num was significantly lower than that in task N-cha (*p* < 0.01), but higher than that in tasks N-obj and N-col (*p*-values < 0.01), while *modularity* in task N-cha was significantly higher than that in tasks N-obj and N-col (*p*-values < 1 × 10^−4^). However, there was no significant difference between tasks N-col and N-obj in *modularity* (*Z* = 2,330, *p* = 1.0).
(8) Community Number: *The community number* in task N-num was significantly lower than that in tasks N-cha and N-col (*p*-values < 0.01). However, there was no significant difference between other tasks in *community number* (*p*-values > 0.05).
(9)Small-worldness: *Small-worldness* in task N-num was significantly lower than that in tasks N-obj and N-col (*p*-values < 1 × 10^−4^), while *small-worldness* in task N-cha was significantly lower than that in tasks N-obj and N-col (*p*-values < 1 × 10^−4^). However, there was no significant difference between tasks N-num and N-cha (*Z* = 2,548, *p* = 0.66), as well as that in N-col and N-obj (*Z* = 2,727, *p* = 0.29), in *small-worldness*.

**Table 1:** Difference between different RAN tasks for each of the topological parameters (M ± SD).

**Topological parameters**	**N-num**	**N-cha**	**N-obj**	**N-col**	***x*** **^2^(3)**	***p***	**Kendall's *W***
Average degree	23.90 ± 5.60	33.00 ± 6.63	54.00 ± 12.30	56.50 ± 14.00	265.82	<1 × 10^−4^	0.90
Network diameter	15.80 ± 2.29	15.70 ± 2.15	14.70 ± 1.58	15.10 ± 1.36	24.39	<1 × 10^−4^	0.08
Characteristic path length	6.07 ± 0.69	6.04 ± 0.59	5.63 ± 0.42	5.65 ± 0.38	65.52	<1 × 10^−4^	0.22
Clustering coefficient	0.72 ± 0.04	0.75 ± 0.03	0.72 ± 0.03	0.73 ± 0.03	47.44	<1 × 10^−4^	0.16
Global efficiency	0.24 ± 0.02	0.24 ± 0.01	0.26 ± 0.01	0.25 ± 0.01	134.07	<1 × 10^−4^	0.46
Assortativity coefficient	0.63 ± 0.11	0.68 ± 0.09	0.64 ± 0.10	0.65 ± 0.09	11.58	0.009	0.04
Modularity	0.78 ± 0.02	0.79 ± 0.02	0.77 ± 0.02	0.77 ± 0.02	51.91	<1 × 10^−4^	0.18
Community number	12.90 ± 2.35	14.60 ± 2.76	13.70 ± 3.14	14.40 ± 3.58	18.11	<1 × 10^−3^	0.06
Small-worldness	0.66 ± 0.06	0.66 ± 0.05	0.69 ± 0.04	0.68 ± 0.03	31.53	<1 × 10^−4^	0.11

RAN, rapid automatized naming; N-num, naming of numbers; N-cha, naming of Chinese characters; N-col, naming of colors; N-obj, naming of objects.

### 3.2. Association between topological parameters and traditional eye-tracking metrics

To examine the independence between each of the nine topological parameters and six traditional eye-movement metrics, we carried out a series of rank-based non-parametric linear regression analyses. Table 2 summarizes our results and shows as given below.

**Table 2:** The values *t* corresponding to the independent variables in the regression model for each of the nine topological parameters, with determination coefficient *R*^2^.

**Task types**	**Topological parameters**	**Total time of naming**	**Average fixation duration**	**Fixation counts**	**Average saccade amplitude**	**Saccade counts**	**Regression counts**	***R*** **^2^**
N-num	Average degree	**561.40** ^d^	**−6.51** ^d^	−1.42	0.11	0.01	0.56	0.99
	Network diameter	−0.88	−0.75	0.87	1.23	**2.29** ^a^	**−3.47** ^c^	0.12
	Characteristic path length	−1.30	0.05	0.81	−0.02	1.36	−1.50	0.08
	Clustering coeff.	−0.41	1.81	0.13	0.10	−0.11	0.28	0.06
	Global efficiency	0.68	−0.18	0.24	−0.10	−0.51	0.88	0.11
	Assortativity coeff.	1.21	−0.22	−1.18	−0.15	−0.45	1.12	0.06
	Modularity	**−2.50** ^a^	**4.07** ^d^	1.53	−0.02	−0.21	0.27	0.17
	Community number	1.53	−0.92	−1.41	0.70	1.19	**−2.42** ^a^	0.05
	Small-worldness	1.16	−0.06	−0.82	−0.23	−1.34	1.54	0.06
N-cha	Average degree	**626.56** ^d^	**−8.30** ^d^	**−2.49** ^a^	1.08	−0.35	−0.81	0.99
	Network diameter	−0.36	−0.08	0.78	0.53	0.06	−1.23	0.05
	Characteristic path length	−1.34	0.58	1.93	1.03	−1.54	0.08	0.19
	Clustering coeff.	−0.94	**2.04** ^a^	1.07	0.43	−0.34	0.12	0.10
	Global efficiency	1.12	−0.45	−1.27	−0.57	0.83	0.75	0.22
	Assortativity coeff.	0.16	0.78	0.13	−0.75	0.19	0.25	0.09
	Modularity	−1.25	1.15	1.74	1.39	−1.55	0.39	0.13
	Community number	−1.35	**2.21** ^a^	0.70	−1.83	1.17	−0.26	0.08
	Small-worldness	1.36	−0.69	**−2.11** ^a^	−1.22	1.74	−0.25	0.19
N-obj	Average degree	**586.64** ^d^	**−5.32** ^d^	−0.75	0.04	−0.03	0.08	0.99
	Network diameter	0.00	0.00	0.00	0.00	0.00	0.00	0.00
	Characteristic path length	0.17	−0.11	−0.54	−1.37	1.36	−1.13	0.06
	Clustering coeff.	0.57	0.75	−0.91	−0.14	0.81	−0.62	0.18
	Global efficiency	−0.09	−0.01	0.56	1.20	−1.29	1.35	0.09
	Assortativity coeff.	−0.33	0.54	−0.14	**−2.02** ^a^	1.61	−0.04	0.09
	Modularity	−0.43	1.47	0.12	0.81	−1.49	0.40	0.22
	Community number	−1.11	1.58	1.04	−1.17	0.63	0.09	0.10
	Small-worldness	−0.12	0.02	0.26	1.64	−1.52	1.12	0.10
N-col	Average degree	**809.94** ^d^	**−5.86** ^d^	−0.78	−3.66^c^	1.74	−0.67	0.99
	Network diameter	0.00	0.00	0.00	0.00	0.00	0.00	0.00
	Characteristic path length	1.95	**−2.02** ^a^	**−2.17** ^a^	−0.05	−0.33	−0.23	0.07
	Clustering coeff.	1.28	−0.34	−1.49	−1.04	0.02	−0.06	0.13
	Global efficiency	**−2.02** ^a^	**2.24** ^a^	**2.34** ^a^	0.31	0.23	0.82	0.12
	Assortativity coeff.	0.80	−0.72	−0.80	−1.16	0.37	0.07	0.05
	Modularity	−0.09	0.29	−0.69	−0.81	−0.19	−0.01	0.16
	Community number	−0.81	1.42	0.14	−1.99	−0.67	1.81	0.12
	Small-worldness	**−2.26** ^a^	**2.30** ^a^	**2.34** ^a^	0.33	0.15	0.28	0.06

a Corresponding to p < 0.05.

c Corresponding to p < 1 × 10^−3^.

d Corresponding to p < 1 × 10^−4^.

Variables with correlation were marked with bold.

#### 3.2.1. Task N-num

(1) Average degree was significantly correlated with the total time of naming (*B* = 1.50, *t* = 561.40, *p* < 1 × 10^−4^) and average fixation duration (*B* = −9.45 × 10^−4^, *t* = −6.51, *p* < 1 × 10^−4^), where the regression model was determined with *R*^2^ = 0.99. (2) Network diameter was significantly correlated with saccade counts (*B* = 0.08, *t* = 2.29, *p* = 0.02) and regression counts (*B* = −0.18, *t* = −3.47, *p* < 1 × 10^−3^), where the regression model was determined with *R*^2^ = 0.12. (3) Modularity was significantly correlated with the total time of naming (*B* = −5.48 × 10^−3^, *t* = −2.50, *p* = 0.01) and average fixation duration (*B* = 4.84 × 10^−4^, *t* = 4.07, *p* < 1 × 10^−4^), where the regression model was determined with *R*^2^ = 0.17. (4) Community number was significantly correlated with regression counts (*B* = −0.16, *t* = −2.42, *p* = 0.02), where the regression model was determined with *R*^2^ = 0.05. (5) Other topological parameters were not significantly correlated with traditional eye-tracking metrics (*p*-values > 0.05).

#### 3.2.2. Task N-cha

(1) Average degree was significantly correlated with total time of naming (*B* = 1.51, *t* = 626.56, *p* < 1 × 10^−4^), average fixation duration (*B* = −0.001, *t* = −8.30, *p* < 1 × 10^−4^), and fixation counts (*B* = −0.002, *t* = −2.49, *p* = 0.01), where the regression model was determined with *R*^2^ = 0.99. (2) Clustering coefficient was significantly correlated with average fixation duration (*B* = 3.97 × 10^−4^, *t* = 2.04, *p* = 0.046), where the regression model was determined with *R*^2^ = 0.10. (3) Community number was significantly correlated with average fixation duration (*B* = 0.04, *t* = 2.21, *p* = 0.03), where the regression model was determined with *R*^2^ = 0.08. (4) Small-worldness was significantly correlated with fixation counts (*B* = −0.002, *t* = −2.11, *p* = 0.038), where the regression model was determined with *R*^2^ = 0.19. (5) Other topological parameters were not significantly correlated with traditional eye-tracking metrics (*p*-values > 0.05).

#### 3.2.3. Task N-obj

(1) Average degree was significantly correlated with total time of naming (*B* = 1.50, *t* = 586.64, *p* < 1 × 10^−4^) and average fixation duration (*B* = −1.13 × 10^−3^, *t* = −5.32, *p* < 1 × 10^−4^), where the regression model was determined with *R*^2^ = 0.99. (2) Assortativity coefficient was significantly correlated with average saccade amplitude (*B* = −5.0 × 10^−3^, *t* = −2.02, *p* = 0.046), where the regression model was determined with *R*^2^ = 0.09. (3) Other topological parameters were not significantly correlated with traditional eye-tracking metrics (*p*-values > 0.05).

#### 3.2.4. Task N-col

(1) Average degree was significantly correlated with total time of naming (*B* = 1.50, *t* = 809.94, *p* < 1 × 10^−4^), average fixation duration (*B* = −0.001, *t* = −5.86, *p* < 1 × 10^−4^), and average saccade amplitude (*B* = −0.002, *t* = −3.66, *p* < 1 × 10^−3^), where the regression model was determined with *R*^2^ = 0.99. (2) Path length was significantly correlated with average fixation duration (*B* = −0.004, *t* = −2.02, *p* = 0.046) and fixation counts (*B* = −0.01, *t* = −2.17, *p* = 0.03), where the regression model was determined with *R*^2^ = 0.07. (3) Global efficiency was significantly correlated with total time of naming (*B* = −1.02 × 10^−3^, *t* = −2.02, *p* = 0.046), average fixation duration (*B* = 1.09 × 10^−4^, *t* = 2.24, *p* = 0.03), and fixation counts (*B* = 4.21 × 10^−4^, *t* = 2.34, *p* = 0.02), where the regression model was determined with *R*^2^ = 0.12. (3) Small-worldness was significantly correlated with the total time of naming (*B* = −3.01 × 10^−3^, *t* = −2.26, *p* = 0.026), average fixation duration (*B* = 2.96 × 10^−4^, *t* = 2.30, *p* = 0.024) and fixation counts (*B* = 1.11 × 10^−3^, *t* = 2.34, *p* = 0.02), where the regression model was determined with *R*^2^ = 0.06. (5) Other topological parameters were not significantly correlated with traditional eye-tracking metrics (*p*-values > 0.05).

## 4. Discussion

Recent studies (Constantino et al., 2017; Del Bianco et al., 2021; Hedger and Chakrabarti, 2021; Nayar et al., 2022) have demonstrated the benefits of considering the eye-tracking data as a time series, as opposed to typical eye-movement measures that require the definition of ROIs and ignore dynamic aspects. It is conceivable to expect that the performance would be enhanced if a non-linear time series analysis technique could be adopted. This study proposed a non-linear eye-movement analysis method from the perspective of the network domain, which constructed a complex network (referred to as GCN) from gaze time series during RAN. This suggests that eye-movement data (responding to gaze behavior) during RAN could be analyzed by computing the topological parameters of GCN. In this way, the proposed method established a link between eye-movement analysis and complex network analysis, much like the Fourier transforms did for time-domain and frequency-domain research. As far as we know, this is the first time to report such an idea, which illustrates a novel study direction for eye-movement analysis.

### 4.1. Structural features of GCN

Small-world architecture (Watts and Strogatz, 1998; Bassett and Bullmore, 2016) may inherit crucial characteristics of many complex systems and, thus, has been significant in the understanding of network sciences, particularly in the study of brain networks (Bullmore and Bassett, 2011). Numerous studies (Bassett and Bullmore, 2016) have been conducted to address the following issues: (i) how to build a small-world network (Watts and Strogatz, 1998); (ii) how to determine whether a network has small-world architecture (Bassett and Bullmore, 2016); and (iii) how to use the difference of small-world index at the group or/and individual level in the understanding of real systems [e.g., brain networks (Bassett and Bullmore, 2016)]. Findings (refer to Figure 3I) showed that SW > 0.4 in all cases. This suggests that GCN may possess “small-world” architecture in all RAN tasks (Bassett and Bullmore, 2017). This “small-world” architecture of GCN is characterized by a distinctive combination of high clustering coefficient and short characteristic path length, which supports high local and global efficiency in information communication and maintains low wiring costs (i.e., sparse connections; Latora and Marchiori, 2001). Such a conception of an “economic small-world” could be interpreted as the biological evolution supporting the optimal GCN structure for TD children.

It is well-known that by randomly adding a few long-range connections to a regular network, one can set up a small-world network (Watts and Strogatz, 1998; Bassett and Bullmore, 2016). However, the current study sought to investigate an inverse issue, i.e., what infrastructure or unit allows the occurrence of small-worldness (i.e., small-world property) in real networks (e.g., GCN in the current study). Small-worldness is typically accompanied by the presence of a community structure or hub nodes, according to earlier studies (Bullmore and Sporns, 2009; He and Evans, 2010; Meunier et al., 2010). This inference may again be supported by our finding (refer to Figure 3G) that GCN in each RAN task has a community structure. Given the existence of community architecture, GCN has intensive intra-community connections and sparse inter-community connections. These sparse inter-community connections (acting as shortcuts) may reduce the characteristic path length of the network and improve global efficiency, while these intensive intra-community connections may boost local clustering and hence facilitate information specialization within a particular community. Therefore, the best balance of information integration and segregation may be ensured by such a well-organized community structure, which would also support the small-world configuration in GCN.

This study assessed the assortativity coefficient which quantifies the tendency of nodes to preferentially connect to nodes with a similar degree (degree-degree correlations). According to our findings (refer to Figure 3F), GCN in each RAN task was regarded as an assortative network with a positive degree–degree correlations because AC > 0. This suggests that high-degree and low-degree nodes in GCN tend to attach to other high-degree and low-degree nodes, respectively. Additionally, when the assortativity coefficient of GCN decreases, this order tends to deteriorate, and some nodes start establishing new connections with nodes with less similar degrees to their own degrees. Given that the assortativity coefficient in non-alphanumeric GCN is lower than that in alphanumeric GCN, GCN in non-alphanumeric RAN tasks may be more likely to worsen nodes' propensity to link to other nodes preferentially than in alphanumeric RAN tasks. It should be noted that reduced assortativity in brain diseases (such as Alzheimer's disease, Parkinson's disease, epilepsy, and depression) was observed (Bialonski and Lehnertz, 2013; Wagner et al., 2019; Conti et al., 2022), in which a complex network was built from brain imaging time series. Therefore, it makes sense to speculate that the assortativity coefficient may comprehend and even recognize the distinction between healthy subjects and patients (e.g., dyslexia).

### 4.2. Mechanism to support small-world architecture and modularity

Gaze behavior consists of three events (Rayner, 1998; Engbert et al., 2002; Bhargavi and Prabha, 2020), namely, fixations, saccades, and regressions. Fixations are recognized, when a gaze is held for at least 100 ms (Harezlak and Kasprowski, 2019). Saccades are very rapid movements, during which the eyes change position to reach another fixation point. Regressions occur when there are backward eye movements toward previously visited items. Based on the observations made above, it is clear to draw the conclusion that fixations can be considered as clustering of eye gazes, saccades as links between fixations, and regressions as sparse long-distance connections between gazes. Hence, this physical mechanism of eye movements may essentially support the presence of community structure in GCN, where intra-community connections are intensive but intra-community connections are sparse. It should be noted that the number of fixations is often higher than the actual number of communities, and one community may typically host many fixations.

For each RAN task, participants were instructed to scan and name the 5 × 10 matrix of objects serially from left to right in each row, and from top to bottom for all rows. This eye movement pattern theoretically resembles eyeballs “walking” on a lattice of gazes. On the contrary, recent studies (Haworth et al., 2015; Harezlak and Kasprowski, 2019; Mohammadhasani et al., 2020) suggested that eye movements might exhibit fractal and chaotic characteristics. These seemingly *random* eye movements, along with an ocular “walk” on a lattice, may provide evidence for the existence of small-world architecture (Watts and Strogatz, 1998; Bassett and Bullmore, 2016). This notion is strongly supported by our finding that GCN in each RAN task displayed small-world architecture (refer to Figure 3I).

### 4.3. Influence of different RAN tasks

This study conducted six pairs of comparisons between four RAN tasks and examined how different tasks affected the topological properties of GCN. Findings (refer to Figure 3) showed that: (i) Average degree and modularity could not indicate the difference between the pair of tasks N-obj and N-col, but they might reflect the difference between each of the other five pairs; (ii) Characteristic path length, global efficiency, and small-worldness could not indicate the difference between the pair of tasks N-obj and N-col, as well as the pair of tasks N-num and N-cha, but they might reflect the difference between each of the other four pairs; (iii) Each of the three pairs of RAN tasks' differences might be reflected by network diameter and clustering coefficient; and (iv) Each of the two pairs of RAN tasks' differences might be reflected by assortativity coefficient and community number.

Additionally, five topological parameters (i.e., average degree, clustering coefficient, assortativity coefficient, modularity, and community number) could reflect the difference between tasks N-num and N-cha. Only one topological parameter (i.e., network diameter) was able to distinguish the difference between tasks N-obj and N-col, but the other eight parameters were unable to do so.

Generally, RAN tasks (Bowers and Wolf, 1993; Kail et al., 1999; Wiig et al., 2000; Stainthorp et al., 2010; American Psychiatric Association, 2013; Decker et al., 2013; Georgiou et al., 2013; Powell et al., 2014; Hjetland et al., 2017; Akhand et al., 2019; Ullman et al., 2020) can be grouped into two categories, namely, alphanumeric and non-alphanumeric RAN tasks. In particular, naming of numbers, letters, words, or Chinese characters belongs to alphanumeric RAN tasks, while naming of colors or objects falls under the category of alphanumeric RAN tasks. Alphanumeric RAN might require mainly phonological processing, i.e., the corresponding verbal codes of these stimuli are readily accessible at the surface level (Donker et al., 2016), while non-alphanumeric RAN seems to demand additional steps and require conceptual processing to establish meaning and subsequently the selection of the appropriate name code, before phonological processing results in articulating a response (Donker et al., 2016). These additional cognitive stages suggest that non-alphanumeric RAN may often need longer naming time, longer fixation duration, and higher fixation counts when compared to alphanumeric RAN. This suggests that GCN in non-alphanumeric RAN may typically have more nodes than GCN in alphanumeric RAN (as a result of the occurrence of more gazes). However, even in such a circumstance, GCN in non-alphanumeric RAN still has a higher average degree, a smaller lower network diameter, and a shorter characteristic path length than that in alphanumeric RAN. This fact supports the hypothesis that GCN in non-alphanumeric RAN might have a more ideal architecture than that in alphanumeric RAN. Accordingly, when compared to alphanumeric RAN, GCN in non-alphanumeric RAN had a higher average degree, global efficiency, and small-worldness, but it also had a lower network diameter, characteristic path length, clustering coefficient, and modularity. The mechanism driving the structural difference between alphanumeric and non-alphanumeric RAN would merit more investigation in future studies.

### 4.4. Association between network properties and traditional metrics

This study carried out a series of rank-based non-parametric linear regression analyses to examine the independence between each of the nine topological parameters and six traditional eye-movement metrics. In all RAN tasks, our results (refer to Table 2) demonstrated that the average degree was dependent on six traditional eye-movement metrics, particularly total naming time, average fixation duration, and fixation counts, with a high determination coefficient *R*^2^ = 0.99; however, the other eight topological parameters were largely independent of these traditional metrics.

Rather than emphasizing the superiority of our technology over traditional eye-tracking metrics, this study attempted to provide a novel gaze time series analysis method to measure gaze behavior from the perspective of the network domain. As noted, the majority of topological parameters might be rather independent and impossible to be predicted using traditional eye-movement metrics. This suggests that our technique might be considered an important supplement to traditional eye movement analysis. It should be noted that one topological parameter (i.e., network diameter) could be utilized to differentiate between tasks N-obj and N-col but the six conventional eye-movement metrics could not.

### 4.5. Network constructed from time series

Many physical systems can be modeled as complex networks (Butts, 2009; Wu et al., 2018; Zhou et al., 2018) by using a proper definition of nodes and edges, which may usually enhance our understanding of their structure and functions. Recent studies have illustrated a promising research direction, which converted time series into a complex network (Zhang and Small, 2006; Lacasa et al., 2012). As an illustration, Zhang and Small (2006) assigned each cycle of a time series to a node and used temporal correlation measures to establish edges (i.e., connections between nodes). They sought, for the first time, to set up a linkage between time series and topological features. They specifically showed that noisy periodic time series could be mapped into random networks, while chaotic time series could be mapped into networks with small-world or/and scale-free features. However, their solution could only be applied to pseudoperiodic time series (Lacasa et al., 2012). In order to overcome this problem, Lacasa et al. (2012) introduced a visibility graph approach that may be used to analyze any type of one-dimensional time series. They showed that periodic time series can be converted into regular networks, random time series can be converted into random networks, and fractal time series can be converted into scale-free networks.

Even though the visibility graph approach offers a promising paradigm for graph-based time series analysis, it can only be applied to one-dimensional time series. Hence, more research is needed to extend the visibility graph approach from one-dimensional time series to multivariate time series. The current study was precisely in this line and made a novel suggestion to map a two-dimensional gaze time series in a complex network in the context of RAN. Clearly, the applicability of the concept of transforming time series into complex networks was further extended by our study.

### 4.6. Cross-cultural or cross-language effects

Researchers should typically use a Chinese adaptation of RAN rather than the original English form when studying developmental dyslexia in Chinese. It should be noted that there are some notable discrepancies between the two versions as a result of Chinese characters' unique characteristics. Typical discrepancies were listed as follows: (i) Chinese characters not only have shape and sound attributes like English letters but also represent meaning; (ii) Chinese characters have no clear form-to-sound conversion rules, thus, readers need to remember the pronunciation of Chinese characters; and (iii) The visual complexity of Chinese characters is much higher than that of English letters. Consequently, when compared to the original RAN, the Chinese adaptation may have higher cognitive complexity and thus activate a wider range of brain regions (Liao et al., 2015; Peng et al., 2017). To extend the application of RAN to developmental dyslexia in Chinese, a Chinese adaptation of RAN (i.e., the C-RAN) was suggested by substituting Chinese characters (highly frequently used) for English letters. Even though the current study did not demonstrate the performance of the C-RAN, it is conceivable to expect that it could be more appropriate than the original RAN for assessing developmental dyslexia in Chinese.

Georgiou et al. (2016) sought to understand the role of RAN in reading fluency and spelling across three languages (i.e., Chinese, English, and Finnish). They showed that: (i) RAN predicted reading fluency equally well across languages; and (ii) RAN did not exert a significant direct effect on spelling, and a substantial proportion of its predictive variance was mediated by phonological processing (in Chinese and Finnish) and orthographic processing (in English). Their findings demonstrated both the cross-cultural consistency and inconsistency which might be reflected by RAN.

Recent studies (Nayar et al., 2021, 2022) examined the cultural effects on gaze behavior. For instance, a cross-cultural study (Nayar et al., 2021) discovered that children (with or without autism) from Hong Kong typically required longer eye-voice spans and more fixations than their counterparts from the US. This finding showed that cross-cultural inconsistency influenced gaze behavior during RAN, which may be captured by eye-movement metrics.

### 4.7. Potential applications and future research

Rapid automatized naming has been widely applied to evaluate several reading-related abilities, such as processing speed (Kail et al., 1999), visual processing ability (Stainthorp et al., 2010), or serial processing ability (Georgiou et al., 2013). It has also been interpreted as an index of word-specific “orthographic” and/or word-specific phonological knowledge (Bowers and Wolf, 1993; Decker et al., 2013; Powell et al., 2014). Therefore, the suggested method can be employed to assess reading-related skills, as well as to aid in the diagnosis of several cognitive abnormalities, including dyslexia (Goswami, 2015; Åvall et al., 2019; Georgiou and Parrilla, 2020), specific language impairment (Snowling and Melby-Lervag, 2016), attention deficit/hyperactivity disorder (ADHD; Tannock et al., 2000), learning disabilities, and autism spectrum disorder (ASD; Hogan-Brown et al., 2014; Zhao et al., 2019). In particular, the suggested technique can be applied to understand the difference in topological parameters in GCN during cognitive tasks between TD children and children with developmental disorders (e.g., dyslexia, specific language impairment, ADHD, and ASD). It would also deserve to examine whether this difference would be affected by age and gender.

Additionally, our technique can essentially be considered as a network-domain measure method to evaluate an individual's RAN abilities. The normative data can be established by calculating the 5, 10, 15, 25, 50, 75, and 90th centiles as grade-specific reference values for each topological parameter in GCN, similar to what we did in our previous research (Xie et al., 2022). Our method could, thus, be applied to assist in the diagnosis of RAN-related cognitive deficits (e.g., dyslexia) by choosing appropriate cut-off values for topological parameters. This notion has been illustrated in our recent study (Xie et al., 2022), which found that attention-related skills might be used to screen for learning difficulties by using the 5th centile as the cut-off value.

### 4.8. Strengths and limitations

Strengths of the current study included that: (i) it conducted a network-domain evaluation of gaze behavior using two-dimensional time series (i.e., temporal-spatial information of eye gazes), in contrast to the visibility graph approach (Lacasa et al., 2012) using one-dimensional time series; and (ii) it did not require manually setting ROIs, which is different from typical eye-tracking analysis. However, there are also some limitations and future studies worth noting. First, this study investigated only monolingual Chinese Grade 6 children (older children). Our decision was based on the fact that by that time, RAN would become automatic (Georgiou and Stewart, 2013), potentially stabilizing its relationship with reading. However, RAN abilities may generally change with age (van den Bos et al., 2002) and depend on the language (e.g., Araújo et al., 2015). This implies that the current single-grade study may miss the opportunity to document developmental changes in RAN abilities across languages. Second, previous studies showed that: (i) Girls have a faster cognitive and social development up to the end of adolescence than boys of the same age (Gur et al., 2012; Xie et al., 2022); (ii) Gender differences may play a significant role in the evaluation of neurological and psychiatric disorders; (iii) The research on learning disabilities and ADHD supports a higher prevalence in boys (Yong and Mcintyre, 1992; Retz-Junginger et al., 2010; Rucklidge, 2010; Williamson and Johnston, 2015); and (iv) Age and gender may combine to affect cognitive abilities (Xie et al., 2022). However, as a limitation, this study did not consider the influence of gender on gaze behavior. In particular, the current study did not adequately capture the gender effect on developmental alterations in RAN abilities at different ages. Finally, this study did not analyze the influence of IQ on gaze behavior during RAN, even though IQ might affect an individual's RAN abilities (Wolff, 2014; Nayar et al., 2021). It should be noted that after controlling the influence of IQ, RAN might predict reading speed (Wolff, 2014). The aforementioned flaws would be discussed in future research.

## 5. Conclusion

This article suggested a new method to measure gaze behavior during RAN from the perspective of the network domain, which mapped gaze time series during RAN into a complex network (referred to as GCN). Findings showed that GCN in each RAN task was assortative and possessed “small-world” and community architecture for TD children. Those topological properties would be applied to evaluate an individual's RAN abilities, as well as to aid in the diagnosis of cognitive abnormalities (e.g., dyslexia) by setting appropriate cut-off values. The influence of different tasks on the topological properties of GCN was examined. The time series analysis methods, cultural effects, potential applications, strengths, and limitations were discussed as well. As the eye-tracking method is fundamental for psychological research, the suggested technique may have the potential to be used in a very broad prospect of applications.

## Data availability statement

The raw data supporting the conclusions of this article will be made available by the authors, without undue reservation.

## Ethics statement

The studies involving human participants were reviewed and approved by the Research Ethics Committee at Southeast University. Written informed consent to participate in this study was provided by the participants' legal guardian/next of kin.

## Author contributions

DY and HW developed the idea for the study and did the analyses. HW and FL collected the data. DY wrote the manuscript. All authors contributed to the article and approved the submitted version.
